# Association between alpha-fetoprotein and metabolic syndrome in a Chinese asymptomatic population: a cross-sectional study

**DOI:** 10.1186/s12944-016-0256-x

**Published:** 2016-04-27

**Authors:** Yimin Chen, Ying Zhao, Linmin Feng, Jie Zhang, Juanwen Zhang, Guofang Feng

**Affiliations:** Department of Clinical Laboratory, Zhejiang Provincial Hospital of Traditional Chinese Medicine, The First Affiliated Hospital of Zhejiang Chinese Medical University, Youdian Road #54, Hangzhou, 310006 China; Department of Clinical Laboratory, The First Affiliated Hospital of Zhejiang University School of Medicine, Qingchun Road #79, Hangzhou, 310003 China; The Affiliated Women’s Hospital of Zhejiang University School of Medicine, Xueshi Road #1, Hangzhou, 310006 China

**Keywords:** Alpha-fetoprotein, Metabolic syndrome, Fatty liver disease

## Abstract

**Background:**

Metabolic syndrome is closely associated with an increased risk for fatty liver disease morbidity and mortality. Recently, studies have reported that participants with fatty liver disease have higher serum alpha-fetoprotein levels than those without. We investigated the association between alpha-fetoprotein levels and the prevalence of metabolic syndrome in a Chinese asymptomatic population.

**Methods:**

A cross-sectional study was performed with 7 755 participants who underwent individual health examinations. Clinical and anthropometric parameters were collected and serum alpha-fetoprotein levels and other clinical and laboratory parameters were measured. Logistic regression analysis was used to examine associations between alpha-fetoprotein and metabolic syndrome.

**Results:**

Participants with metabolic syndrome had significantly higher (*p* < 0.001) alpha-fetoprotein levels than those without, though all alpha-fetoprotein levels were within the reference interval. The association between the components of metabolic syndrome (central obesity, elevated blood pressure, elevated triglycerides, reduced high-density lipoprotein cholesterol, and elevated fasting plasma glucose) and alpha-fetoprotein levels was evaluated. Alpha-fetoprotein levels in the elevated triglycerides, reduced high-density lipoprotein cholesterol, and elevated fasting plasma glucose groups were significantly different (*p*=0.002, *p* < 0.001, *p*=0.020) compared with alpha-fetoprotein in the normal triglycerides, high-density lipoprotein cholesterol, and fasting plasma glucose groups. Logistic regression analyses showed an association between alpha-fetoprotein levels and increased risk for metabolic syndrome, the presence of reduced high-density lipoprotein cholesterol, and elevated fasting plasma glucose, but not with obesity, elevated blood pressure, or triglycerides.

**Conclusions:**

These results suggest a significant association between alpha-fetoprotein and metabolic syndrome.

## Background

Metabolic syndrome (MS) is characterized by a number of metabolic risk factors including abdominal obesity, impaired glucose metabolism, dyslipidemia, and hypertension [1–3]. MS is also associated with the development of diabetes mellitus, cardiovascular disease, and non-alcoholic fatty liver disease (NAFLD) [1, 4, 5]. The prevalence of MS is increasing worldwide. In China, Gu et al. [6] reported that the prevalence of MS was 9.8 % in men and 17.8 % in women in 2005. Zhou et al. [7] conducted a cross-sectional survey in 14 provinces in China and reported that the prevalence of MS was 22.1 % in men and 25.8 % in women in 2014. Insulin resistance (IR) and chronic inflammation appear to be central mechanisms underlying the pathophysiology of MS [8].

Alpha-fetoprotein (AFP) is a single-stranded glycoprotein primarily produced by the fetal liver and yolk sac. The level of AFP declines rapidly after birth and remains at a low level throughout the life of a person in the normal population [9–11]. AFP levels are reactivated in liver regeneration and hepatocarcinogenesis, which occur in hepatocellular carcinoma, chronic liver disease, acute or chronic viral hepatitis, and gonadal tumors [12–14]. Elevated serum AFP levels have been used mainly to predict the development of hepatocellular carcinoma, including large tumor size, advanced or metastatic stages, portal vein thrombosis, and postoperative recurrence [15]. Recently, Xu et al. [16] investigated the association between serum AFP and fatty liver disease (FLD) in a population of 9 800 people undergoing health examinations and found that participants with FLD had higher AFP levels than those without. Additionally, Babalı et al. [12] found that patients with NAFLD had higher AFP levels than those without. AFP levels become increasingly elevated as the grade of liver steatosis increases, which suggests that AFP level monitoring might help clinicians to treat NAFLD.

The presence of hepatic inflammation, steatosis, and/or fibrosis may be the underlying cause of increased serum AFP levels in FLD patients with severe fatty liver [12, 16]. Because MS is closely associated with an increased risk for FLD morbidity and mortality [16], we investigated the associations between serum AFP and the prevalence of MS in a Chinese asymptomatic population.

## Methods

### Patients

This study included 8 695 consecutive participants (4 609 men and 4 086 women) who underwent individual health examinations that included a physical examination and clinical laboratory tests at the Health Care Centre of the First Affiliated Hospital of Zhejiang University School of Medicine between April and October 2014. All participants underwent serum AFP and biochemical parameter analyses. Patients were excluded if they exhibited any of the following conditions: viral hepatitis (*n*=403), autoimmune hepatitis (*n*=6), renal insufficiency (*n*=89), chronic liver disease (*n*=105), cancer (*n*=65), elevated tumor markers (*n*=87), pregnancy (*n*=50), and recent infection (*n*=135). The remaining 7 755 participants (4 209 men and 3 546 women) were enrolled in the study. Informed consent was obtained from all participants and the study was approved by the ethics committee of the First Affiliated Hospital of the Medical College of Zhejiang University of China.

### Clinical and anthropometric parameters

Waist circumference (WC), weight, height, systolic blood pressure (SBP), and diastolic blood pressure (DBP) were measured. Body mass index (BMI) was calculated as weight in kilograms divided by height in meters squared. Those with alcohol consumption >140 g/week for men and >70 g/week for women were categorized as drinkers, and those who smoked one or more cigarettes daily for at least 6 months were categorized as smokers.

### Laboratory techniques

Laboratory examinations were conducted in the morning after an overnight fast. Alanine aminotransferase (ALT), aspartate aminotransferase (AST), gamma-glutamyltransferase (γ-GT), triglycerides (TG), total cholesterol (Tch), high-density lipoprotein cholesterol (HDL-c), low-density lipoprotein cholesterol (LDL-c), fasting plasma glucose (FPG), and creatinine (Cr) were assessed using an automatic biochemical analyzer (Hitachi 7600; Hitachi, Tokyo, Japan) with GmbH reagents (Roche Diagnostics, GmbH, Mannheim, Germany). Hemoglobin A1c (HbA1c) was assessed using a high-performance liquid chromatography analyzer (G8-90SL; Tosoh Corp., Tokyo, Japan) with Tosoh reagents. Analysis of AFP was performed using an automated chemiluminescence analyzer (Architect ci8200; Abbott Laboratories, Abbott Park, IL) with Abbott reagents.

### Diagnostic criteria for MS

According to the International Diabetes Federation guidelines [17], MS is defined as having three or more of the following components: (i) WC >90 cm for Chinese men and >80 cm for Chinese women; BMI ≥25 kg/m^2^; (ii) TG ≥1.7 mmol/L, or taking specific treatment for lipid abnormality; HDL-c <1.03 mmol/L for men and <1.29 mmol/L for women; (iii) SBP ≥130 mmHg, or DBP ≥85 mmHg, or treatment for previously diagnosed hypertension; (iv) FPG ≥5.6 mmol/L or previously diagnosed type 2 diabetes.

### Statistical analyses

Statistical analyses were performed using SPSS version 16 (SPSS, Chicago, IL). Data that were normally distributed are reported as mean ± standard deviation and data that had a skewed distribution are reported as median and range. Differences between two groups were analyzed using the Student’s *t*-test or the Mann–Whitney *U* test. Differences among multi-groups were analyzed using one-way analysis of variance or the Kruskal–Walls H test. Spearman correlation analysis was used to examine correlations between serum AFP levels and clinical and laboratory parameters. Univariable and multivariable logistic regression was used to examine associations between AFP and MS. All statistical tests were two-tailed and a *p*-value <0.05 was considered statistically significant.

## Results

### Clinical characteristics of participants

Demographic and biochemical characteristics of study participants are shown in Table 1. There were 7 755 participants included in the study and 1 512 (19.5 %) fulfilled diagnostic criteria for MS. Patients with MS had significant differences in age, WC, BMI, SBP, DBP, ALT, AST, γ-GT, TG, Tch, HDL-c, LDL-c, FPG, HbA1c, Cr, AFP, prevalence of drinking, and FLD compared with patients without MS. The characteristics of all participants according to the number of components of MS are shown in Table 2. We also found significant differences in WC, BMI, SBP, DBP, ALT, AST, γ-GT, TG, Tch, HDL-c, LDL-c, FPG, HbA1c, Cr, AFP, prevalence of drinking, and FLD among these five groups. Participants with MS in groups with three and four MS components had higher age, WC, BMI, SBP, DBP, ALT, AST, γ-GT, TG, Tch, FPG, LDL-c, HbA1c, Cr, AFP, prevalence of drinking, and FLD, and lower HDL-c compared with patients in groups without MS or with 0–2 MS components. Notably, in groups MS 0 to MS 4, there were significantly higher AFP levels observed in participants with MS (*p* < 0.001), although AFP levels were within the normal laboratory reference interval (0–20 ng/mL).

**Table 1:** Demographic and biochemical characteristics of the study participants

Variable	Without MS	With MS	*P* value
n	6243	1512	
Age (yr)	43 (18–87)	51 (26–82)	<0.001
Sex (M/F)	3387/2856	822/690	0.937
WC (cm)	82 (58–123)	91 (64–115)	<0.001
BMI (kg/m^*2*^)	23.2 (15.6–36.9)	26.1 (17.3–35.6)	<0.001
SBP (mmHg)	123 ± 17	143 ± 15	<0.001
DBP (mmHg)	75 ± 11	87 ± 10	<0.001
ALT (U/L)	18 (4–188)	24 (4–193)	<0.001
AST (U/L)	18 (4–188)	21 (7–127)	<0.001
γ-GT (U/L)	21 (3–429)	34 (8–816)	<0.001
TG (mmol/L)	1.13 (0.3–17.1)	1.99 (0.38–16.09)	<0.001
Tch (mmol/L)	4.69 (2.02–10.3)	4.92 (2.62–9.0)	<0.001
HDL-c (mmol/L)	1.27 (0.6–2.9)	1.02 (0.6–2.4)	<0.001
LDL-c (mmol/L)	2.59 (0.75–6.76)	2.66 (1–6.12)	<0.001
FPG (mmol/L)	4.78 (3.45–16)	5.19 (3.87–13)	<0.001
Cr (mmol/L)	67 (29–119)	74 (36–118)	<0.001
HbA1c (%)	5.5 (3.5–11.6)	5.8 (4.2–13)	<0.001
AFP (ng/ml)	2.4 (0.5–19.1)	2.7 (0.8–16.6)	<0.001
Drinker (%)	16.0	29.6	<0.001
Smoker (%)	27	25.7	0.314
FLD (%)	29.0	62.3	<0.001

*WC* waist circumference, *SBP* systolic blood pressure, *DBP* diastolic blood pressure, *BMI*, body mass index, *ALT* alanine aminotransferase, *AST* aspartate aminotransferase, *γ-GT* gamma-glutamyltransferase, *TG* triglycerides, *Tch* total cholesterol, *HDL-c* high-density lipoprotein cholesterol, *LDL-c* low-density lipoprotein cholesterol, *FPG* fasting plasma glucose, *Cr* creatinine, *HbA1c* hemoglobin A1c, *AFP* alpha-fetoprotein, *FLD* fatty liver disease

**Table 2:** Clinical characteristics of the study participants grouped by the number of metabolic syndrome components

Variable	No of components of MS					*P* value
	MS 0	MS 1	MS 2	MS 3	MS 4	
n	1335	2598	2310	1179	333	<0.001
Age (yr)	43 (18–78)	44 (18–87)	47 (18–82)	51 (26–79) #*$	52 (31–82) #*$△	<0.001
Sex (M/F)	735/600	1296/1302	1356/954	621/558$	201/132*△	<0.001
WC (cm)	77 (58–90)	80 (60–123)	88 (60–116)	90 (64–115) #*$	92 (81–115) #*$△	<0.001
BMI (kg/m2)	21.6 (15.6–25)	22.7 (15.9–36.9)	25.1 (17.4–36.7)	26.1 (17.3–35.6) #*$	26.3 (22.9–32.3) #*$△	<0.001
SBP (mmHg)	113 ± 9	119 ± 14	132 ± 17	140 ± 15#*$	150 ± 14#*$△	<0.001
DBP (mmHg)	69 ± 7	73 ± 10	81 ± 12	86 ± 10#*$	90 ± 9#*$△	<0.001
ALT (U/L)	15 (4–69)	17 (5–188)	20 (4–151)	24 (4–123) #*$	30 (10–193) #*$△	<0.001
AST (U/L)	18 (10–48)	19 (9–114)	20 (10–132)	21 (7–62) #*$	23 (11–67) #*$△	<0.001
γ-GT (U/L)	16 (3–377)	20 (5–306)	24 (4–429)	31 (8–816) #*$	50 (10–485) #*$△	<0.001
TG (mmol/L)	0.88 (0.31–1.69)	1.16 (0.3–9.68)	1.3 (0.32–17.1)	1.9 (0.8–16.09) #*$	2.37 (0.81–13.76) #*$△	<0.001
Tch (mmol/L)	4.65 (2.92–7.89)	4.69 (2.13–8.49)	4.71 (2.02–10.3)	4.86 (2.62–9) #*$	5.18 (2.8–8.23) #*$△	<0.001
HDL-c (mmol/L)	1.43 (1.03–2.7)	1.23 (0.57–2.86)	1.17 (0.62–2.27)	1.03 (0.58–2.4) #*$	1.0 (0.67–2) #*$	<0.001
LDL-c (mmol/L)	2.59 (1.26–5.62)	2.59 (0.94–6.32)	2.6 (0.75–6.76)	2.66 (1–6.12) #*$	2.69 (1.35–6.0) #*$	0.001
FPG (mmol/L)	4.73 (3.72–5.57)	4.78 (3.64–13)	4.81 (3.45–16)	5 (3.87–17) #*$	6 (4.44–14) #*$△	<0.001
Cr (mmol/L)	61 (29–100)	67 (33–119)	71 (35–118)	76 (36–114) #*$	72 (44–118) #*$	<0.001
HbA1c (%)	5.5 (3.5–6.1)	5.5 (4.4–10.8)	5.6 (4–11.6)	5.7 (4.2–13) #*$	6.4 (4.7–13) #*$△	<0.001
AFP (ng/ml)	2.3 (0.5–13.2)	2.5 (0.5–11.4)	2.5 (0.5–19.1)	2.7 (0.8–16.6) #*$	2.7 (1.1–10.1) #*$	<0.001
Drinker (%)	13.8	15.3	19.9	27.6 #*$	37.0#*$△	<0.001
Smoker (%)	24.3	27.0	28.5	27.2	20.7	0.142
FLD (%)	12.1	22.5	45.5	63.6#*$	60.7#*$	<0.001

*P*-value: comparison among these five groups; # comparison vs. MS 0 group; * comparison vs. MS 1 group; $ comparison vs. MS 2 group; Δ comparison vs. MS 3 group. *WC* waist circumference, *SBP* systolic blood pressure, *DBP* diastolic blood pressure, *BMI* body mass index, *ALT* Alanine aminotransferase, *AST* aspartate aminotransferase, *γ-GT* gamma-glutamyltransferase, *TG* triglycerides, *Tch* total cholesterol, *HDL-c* high-density lipoprotein cholesterol, *LDL-c* low-density lipoprotein cholesterol, *FPG* fasting plasma glucose, *Cr* creatinine, *HbA1c* hemoglobin A1c, *AFP* alpha-fetoprotein, *FLD* fatty liver disease

### Association between MS and serum AFP levels

Serum AFP levels significantly correlated with age, WC, ALT, AST, γ-GT, HDL-c, LDL-c, TG, and Tch (*r*=−0.087, 0.121, 0.058, 0.085, 0.218, 0.113, 0.085, 0.232, and 0.188, respectively; all *p* < 0.05) in participants with MS. In participants without MS, AFP significantly correlated with ALT, AST, γ-GT, LDL-c, TG, and Tch (*r*=0.121, 0.105, 0.212, 0.131, 0.137, and 0.162, respectively; all *p* < 0.05).

**Fig. 1:**
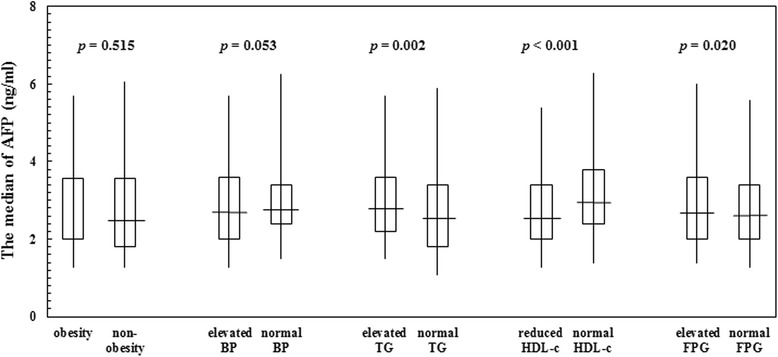
Serum alpha-fetoprotein levels according to characteristics of metabolic syndrome in participants with metabolic syndrome

**Fig. 2:**
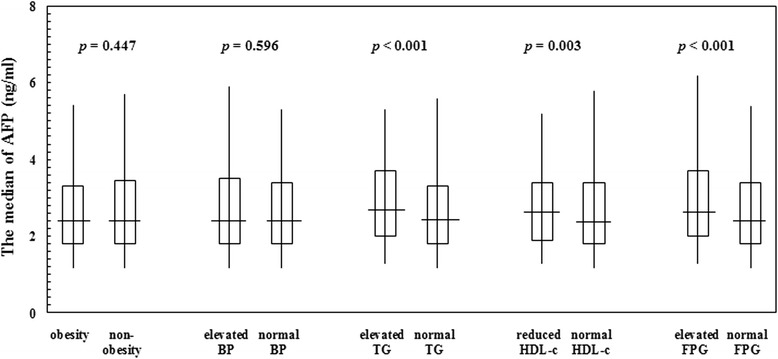
Serum alpha-fetoprotein levels according to characteristics of metabolic syndrome in participants without metabolic syndrome

The components of MS included central obesity, elevated blood pressure, elevated TG, reduced HDL-c, and elevated FPG. The association between the components of MS and serum AFP levels in participants with and without MS are shown in Figs. 1 and 2. AFP levels in elevated TG, reduced HDL-c, and elevated FPG groups were significantly different compared with AFP in normal TG, HDL-c, and FPG groups in all participants.

**Fig. 3:**
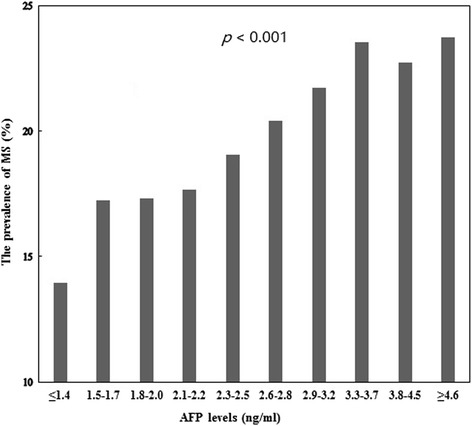
Association between serum alpha-fetoprotein levels and the prevalence of metabolic syndrome

Participants were divided into 10 groups according to deciles of serum AFP levels. The association between the 10 groups and the prevalence of MS are shown in Fig. 3. Across increasing serum AFP deciles, the prevalence of MS increased (*p* < 0.001).

### Risk factors for the presence of MS

Univariate and multivariate logistic regression analyses was used to analyze the risk factors for MS in these asymptomatic participants. In the univariate regression model, serum AFP levels were associated with an increased risk for MS (odds ratio (OR): 1.072, 95 % confidence interval (95 % CI): 1.037–1.107, *p* < 0.001; Table 3). After adjusting for age, WC, BMI, SBP, DBP, ALT, AST, γ-GT, TG, Tch, HDL-c, LDL-c, FPG, HbA1c, Cr, prevalence of drinking, and FLD using multivariate logistic analysis, AFP levels were also associated with an increased risk for MS (OR: 1.066, 95 % CI: 1.007–1.129, *p*=0.028; Table 3). The association between AFP and the presence of components of MS in the asymptomatic participants was also analyzed. The results did not show an association between AFP levels and the presence of central obesity (OR: 1.009, 95 % CI: 0.958–1.062, *p*=0.743), elevated blood pressure (OR: 1.032, 95 % CI: 0.992–1.073, *p*=0.119), or elevated TG (OR: 1.02, 95 % CI: 0.98–1.062, *p*=0.338). However, AFP levels were associated with the presence of reduced HDL-c (OR: 0.946, 95 % CI: 0.907–0.986, *p*=0.008) and elevated FPG (OR: 1.053, 95 % CI: 1.012–1.096, *p*=0.010).

**Table 3:** Univariable and multivariable regression analyses for variables associated with metabolic syndrome

Variable	univariable		multivariable	
	OR (95 % CI)	*P* value	OR (95 % CI)	*P* value
Age (yr)	1.054 (1.049–1.060)	<0.001	1.038 (1.028–1.049)	<0.001
Sex (male)	0.995 (0.889–1.114)	0.937		
WC (cm)	1.106 (1.098–1.113)	<0.001	1.044 (1.024–1.065)	<0.001
BMI (kg/m^*2*^)	1.345 (1.318–1.373)	<0.001	1.124 (1.062–1.190)	<0.001
SBP (mmHg)	1.067 (1.063–1.071)	<0.001	1.046 (1.037–1.056)	<0.001
DBP (mmHg)	1.094 (1.088–1.100)	<0.001	1.027 (1.013–1.041)	<0.001
ALT (U/L)	1.020 (1.016–1.023)	<0.001	0.989 (0.980–0.998)	0.016
AST (U/L)	1.031 (1.024–1.037)	<0.001	1.022 (1.005–1.04)	0.012
γ-GT (U/L)	1.010 (1.009–1.012)	<0.001	1.005 (1.003–1.008)	<0.001
TG (mmol/L)	1.712 (1.63–1.798)	<0.001	1.192 (1.071–1.326)	<0.001
Tch (mmol/L)	1.377 (1.297–1.462)	<0.001	1.082 (0.816–1.433)	0.585
HDL-c (mmol/L)	0.057 (0.045–0.073)	<0.001	0.032 (0.02–0.052)	<0.001
LDL-c (mmol/L)	1.226 (1.133–1.325)	<0.001	1.081 (0.777–1.504)	0.644
FPG (mmol/L)	1.971 (1.845–2.105)	<0.001	1.722 (1.477–2.008)	<0.001
Cr (mmol/L)	1.027 (1.023–1.031)	<0.001	1.008 (1.001–1.015)	0.028
HbA1c (%)	2.815 (2.506–3.162)	<0.001	1.600 (1.278–2.004)	<0.001
AFP (ng/ml)	1.072 (1.037–1.107)	<0.001	1.066 (1.007–1.129)	0.028
Drinker (yes)	1.848 (1.607–2.126)	<0.001	0.836 (0.652–1.071)	0.156
Smoker (yes)	1.05 (0.914–1.206)	0.492		
FLD (yes)	4.051 (3.602–4.556)	<0.001	1.496 (1.210–1.849)	<0.001

*WC* waist circumference, *SBP* systolic blood pressure, *DBP* diastolic blood pressure, *BMI* body mass index, *ALT* alanine aminotransferase, *AST* aspartate aminotransferase, *γ-GT* gamma-glutamyltransferase, *TG* triglycerides, *Tch* total cholesterol, *HDL-c* high-density lipoprotein cholesterol, *LDL-c* low-density lipoprotein cholesterol, *FPG* fasting plasma glucose, *Cr* creatinine, *HbA1c* hemoglobin A1c, AFP alpha-fetoprotein, *FLD* fatty liver disease

**Fig. 4:**
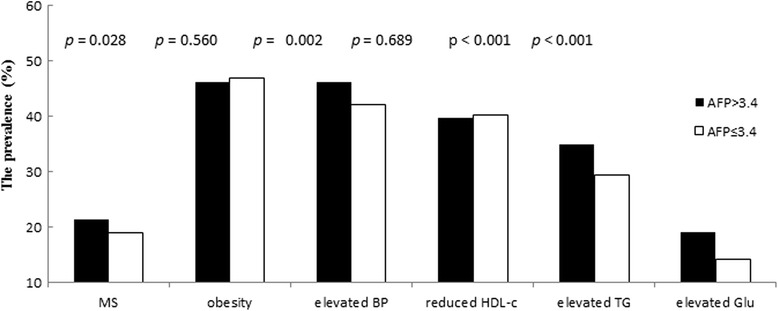
Prevalence of metabolic syndrome and its components according to different serum alpha-fetoprotein levels

Analysis of the association between higher AFP (>3.4 ng/mL) and the prevalence of MS and its components is shown in Fig. 4. The 7 755 participants were divided into two groups according to the 75 % quartile level of serum AFP (3.4 ng/mL). We observed a significantly higher prevalence of MS with the components including elevated BP, elevated TG, and elevated FPG in participants with AFP >3.4 ng/mL compared with those with AFP ≤3.4 ng/mL (Fig. 4).

These results suggest that the presence of reduced HDL-c and elevated FPG may play a major role in the association between AFP and MS, while elevated BP and elevated TG may influence the association.

## Discussion

In this study, serum AFP levels significantly correlated with WC, HDL-c, and TG (*p* < 0.001, *p* < 0.001, and *p* < 0.001, respectively) in participants with MS. Participants with MS had significantly higher AFP levels (*p* < 0.001) than those without MS, although all AFP levels were within the reference interval. We evaluated the association between the components of MS (central obesity, elevated BP, elevated TG, reduced HDL-c, and elevated FPG) and AFP levels, and found that AFP levels in the elevated TG, reduced HDL-c, and elevated FPG groups were significantly different (*p*=0.002, *p* < 0.001, and *p*=0.020) compared with AFP in the normal TG, HDL-c, and FPG groups. Furthermore, we found that in the higher AFP group (>3.4 ng/mL), there was a higher prevalence of MS, elevated BP, elevated TG, and elevated FPG. Logistic regression analyses showed an association between AFP levels and increased risk for MS, reduced HDL-c, and elevated FPG, but no association between obesity, elevated BP, or TG. These results suggest a significant association between AFP and MS (*p*=0.028). Impaired glucose metabolism and dyslipidemia may play a major role in the association between AFP and MS.

Verhagen et al. [18] found that the prevalence of MS was highest in the IR group, and IR increased with the number of MS components. IR is associated with excessive fat accumulation in ectopic tissues, such as the liver, and plays a crucial role in the pathologic manifestations of MS, and is accompanied by elevated BP, elevated TG, reduced HDL-c, and impaired FPG [2, 19]. Some studies previously reported that FLD was more prevalent in patients with MS than in the general population, and FLD may be the hepatic component of MS because MS and FLD have a particularly close relationship [20–23].

The main potential mechanisms accounting for the association between MS and high AFP levels may be IR and hepatic steatosis. Hepatocytes play a major role in glucose homeostasis and can store or produce glucose depending on physical requirements [19]. IR impacts the hepatic glucose homeostatic pathways and leads to the release of free fatty acids from adipose tissue, elevates hepatic production of very-low-density lipoproteins, reduces high-density lipoproteins, and promotes inflammation and endoplasmic reticulum stress [18, 19]. In a study with a large cohort, Porepa et al. [24] found newly diagnosed diabetes in adults with or without pre-existing hypertension, dyslipidemia, or obesity who appeared to be at higher risk for advanced liver disease than isolated hypertension, dyslipidemia, or obesity. In a study by Matsuzaka et al. [25], in which hepatic steatosis was associated with the development of IR, it was remained unclear whether IR leads to hepatic steatosis or whether steatosis enhances IR. Additionally, hepatic steatosis is associated with dyslipidemia and inflammatory markers [20, 21, 25]. Other studies have reported that increased chronic inflammation and oxidative stress in accumulated adipose tissue is an important pathogenic mechanism of obesity-associated MS [26–29]. Another potential mechanism accounting for the association between MS and high AFP levels may be a state of chronic low-level inflammation and oxidative stress.

Serum AFP is generally recognized as an important tumor marker and has specific diagnostic utilities [30]. Continuous elevation of AFP levels up to the pathological range in adults has been associated with hepatocellular carcinoma, gastric cancer, hepatic necrosis, hepatic cirrhosis, acute hepatitis, chronic active hepatitis, ataxia telangiectasia, Wiskott–Aldrich syndrome, and pregnancy [30, 31]. Continuous elevation of serum AFP has rarely been found in participants with no obvious pathology [30, 32].

Adult hepatocytes re-express AFP mainly through three mechanisms: (i) Adult hepatocytes are regarded as functional stem cells and have the inherent capacity for regeneration [33]. When hepatocytes regenerate, AFP levels increase [34]. Hepatocyte proliferation during reparative and regenerating growth is associated with progenitor cell activation and is revealed by observation of rising serum AFP levels, cellular AFP immune expression, and AFP gene expression during hepatocyte division in liver regeneration after chemical injury or partial hepatectomy [33–36]. (ii) DNA damage to hepatocytes induced by oxidative stress results in the activation of transcription factors and induces the expression of proto-oncogenes by DNA methylation, leading to genomic instability and, consequently, hepatocarcinogenesis. Additionally, hepatomas that originate from spontaneously retro-differentiated hepatocytes can express AFP [27, 37, 38]; (c) In very extensive or chronic inflammation liver injury models, when the regenerative capacity of hepatocytes is impeded, reconstitution of the liver occurs through biliary epithelial cells (oval cells) that possess regenerative capacity with multilinear differentiation potential. Biliary epithelial cell proliferation leads to increases in AFP-specific immune expression [34, 39, 40].

Recently, a large sample cross-sectional study by Xu et al. [16] found that participants with FLD had higher AFP levels than those without FLD, and suggested that hepatocyte necrosis and subsequent hepatic regeneration may be responsible for the elevation of serum AFP levels. Babalı et al. [12] found that patients with NAFLD had higher AFP levels, and suggested that hepatic inflammation, regeneration and/or fibrosis may be responsible for the elevation of serum AFP levels in patients with severe fatty liver. We also considered it possible that hepatocyte steatosis and subsequent hepatic regeneration are responsible for the elevation of serum AFP levels in patients with MS. Hepatic steatosis has been reported to be a common histological feature of hepatitis C viral infection [41], and Goldstein et al. [42] found altered hepatocyte–hepatocyte interaction and loss of normal architectural arrangements leading to the elevation of serum AFP levels in patients with chronic hepatitis.

MS is a lifestyle and diet-related chronic non-communicable disease that has become a major burden on global healthcare [38, 43, 44]. Epidemiological studies indicate that nutrition plays a very important role in the development and progression of MS. A diet high in fat, cholesterol, and sugar promotes the redistribution of body fat from peripheral to visceral adipose tissue and affects total body weight [43, 44]. Animal experiments have been reported in which liver tissue sections from rats with diet-induced MS showed increased wet weight, hepatocyte damage, fat vacuoles, fibrosis, collagen deposition, ballooning, and inflammatory cell infiltration [45, 46]. Body fat mass was found to increase in these studies, and impaired glucose tolerance, plasma lipid abnormalities, hyperinsulinemia, and increased liver enzyme activity were observed [45, 46].

Oxidative stress has emerged as a central player in some chronic metabolic diseases and leads to the oxidation of lipids, proteins, and nucleic acids. The above-mentioned pathological liver changes and stages of fibrosis were also found to be associated with oxidative DNA damage [27, 38]. Nishida N et al. found that patients with high serum AFP levels and high degrees of ballooning and inflammatory infiltration had an accumulation of oxidative DNA damage [38]. Furukawa et al. [29] found that increased oxidative stress led to dysregulated production of adipocytokines, and that increased reactive oxygen species production from adipose tissue led to increased oxidative stress in the blood, which hazardously affected the liver, skeletal muscle, and the aorta in MS patients. Kuhlmann et al. and Assimakopoulos et al. observed oval cell proliferation during liver regeneration concomitant with increased AFP expression, with the mechanism being different from AFP expression in hepatocytes [34, 39]. From this previous research and our own observations, we considered that oxidative stress and oval cell proliferation were responsible for the elevation of serum AFP levels in patients with MS.

Our study had some limitations. First, serum insulin levels were not analyzed and homeostasis model of assessment-insulin resistance was not calculated. Our study indirectly indicates a relationship between serum AFP levels and IR, but further research on a direct relationship between AFP and IR needs to be conducted. Second, hepatic inflammation is an important factor in the progression of chronic liver disease and is regulated by chemokines [47]. We did not analyze the effect of chemokines in the relationship between serum AFP levels and IR. Third, our study was a cross-sectional study and no definitive conclusions can be made on causality or temporal associations between AFP and MS.

## Conclusion

In conclusion, the results from this study suggest that participants with MS have significantly higher AFP levels than those without MS. Serum AFP levels were significantly associated with the prevalence of MS, though the linking of serum AFP levels and the prevalence of MS is complicated and requires further prospective investigation.

## Ethical approval and consent to participate

Informed consent was obtained from all participants and the study was approved by the ethics committee of the First Affiliated Hospital of the Medical College of Zhejiang University of China.

## Abbreviations

AFP: alpha-fetoprotein
ALT: alanine aminotransferase
AST: aspartate aminotransferase
Cr: creatinine
DPB: diastolic blood pressure
FLD: fatty liver disease
FPG: fasting plasma glucose
HbA1c: hemoglobin A1c
HDL-c: high-density lipoprotein cholesterol
IR: insulin resistance
LDL-c: low-density lipoprotein cholesterol
MS: metabolic syndrome
NAFLD: non-alcoholic fatty liver disease
OR: odds ratio
SPB: systolic blood pressure
Tch: total cholesterol
TG: triglycerides
WC: waist circumference
γ-GT: gamma-glutamyltransferase
95 % CI: 95 % confidence interval
